# Influence of Different Surface Treatments on the Low-Temperature Degradation of Three Commercial Yttria-Stabilized Tetragonal Zirconia Polycrystal

**DOI:** 10.3390/ma18112543

**Published:** 2025-05-28

**Authors:** Jumei Tian, Huei-Jyuan Liao, Wen-Fu Ho, Hsueh-Chuan Hsu, Shih-Ching Wu

**Affiliations:** 1 Engineering Research Center of Stomatological Biomaterials, Fujian Province University, School of Stomatology of Xiamen Medical College, Xiamen 361023, China; 2 College of Veterinary Medicine, National Chiayi University, No. 300 Syuefu Rd., Chiayi City 600355, Taiwan; pipi324615@gmail.com; 3 Department of Chemical and Materials Engineering, National University of Kaohsiung, Kaohsiung 811726, Taiwan; fujii@nuk.edu.tw; 4 Department of Dental Technology and Materials Science, Central Taiwan University of Science and Technology, Taichung 406053, Taiwan; hchsu@ctust.edu.tw

**Keywords:** zirconia, low-temperature degradation, sandblasting, polishing, acetic acid

## Abstract

Aging of yttria-stabilized tetragonal zirconia polycrystal (Y-TZP) under wet conditions is known as low-temperature degradation (LTD), which is associated with phase change and decreasing mechanical strength. Herein, we studied the effects of different surface treatments on the LTD of three different commercial Y-TZP blocks utilizing CAD/CAM technology, namely, **Cercon^®^, e.max^®^ ZirCAD**, and **Vita In-ceram^®^ YZ**. The blocks were immersed in 4% acetic acid at 80 °C for 0, 7, 14, and 28 days. The effects of surface treatments such as sandblasting and polishing were also examined. The results showed that the monoclinic phase increased with immersion time in all three brands. In **Cercon^®^** blocks, a minimal amount of phase transformation was observed, with the smallest amount of degradation after immersion. Sandblasting and polishing both suppressed phase transformation. After immersion, the mechanical strength exhibited a small decrease with time. Accelerating the evaluation of the LTD of zirconia may effectively help with clinical applications.

## 1. Introduction

In recent years, the development of zirconia has led to increased usage as a prosthetic material in dentistry, due to its excellent biocompatibility, aesthetics, low thermal conductivity, and high flexural strength and toughness [1,2]. Zirconia occurs in three polymorphs, depending on the temperature: monoclinic, *m* (room temperature to 1170 °C); tetragonal, *t* (1170–2370 °C); and cubic, *c* (greater than 2370 °C) [3]. Commercial zirconia dental ceramics with stabilizers such as Y_2_O_3_, CaO, MgO, or CeO_2_ allow for the retention of the tetragonal structure at room temperature [4]. For dental applications, zirconium oxide (ZrO_2_) stabilized with 3 mol% yttrium oxide (Y-TZP) has been widely used [5,6]. Y-TZP presents the phenomenon of phase transition toughening, i.e., stress-induced tetragonal to monoclinic (*t*→*m*) transformation, which can cause volume expansion (4–5%) and arrest the formation and propagation of cracks, leading to high toughness [7]. The *t*→*m* phase transformation can also be induced at comparatively low temperatures, especially in the presence of water. This is commonly known as aging or low-temperature degradation (LTD) [8].

Dental Y-TZP prostheses routinely undergo grinding, polishing, or sandblasting to adjust occlusion or enhance bonding [9]. While some studies suggest that sandblasting improves strength via the induction of compressive stresses [10], others report that it accelerates LTD due to the introduction of surface flaws [11]. Herein, we systematically evaluate how these clinically unavoidable treatments influence LTD, providing actionable insights for prosthodontic workflows.

The ISO 6872 chemical solubility test (4% acetic acid, 80 °C) is standardized for ceramic dissolution, but it is rarely applied in Y-TZP aging studies, which predominantly use autoclaving (134 °C, 2 bar steam) [12]. However, autoclaving fails to replicate oral chemical corrosion (e.g., acidic foods, bacterial byproducts). Our adoption of acetic acid immersion bridges this gap by correlating laboratory aging conditions with clinically relevant degradation pathways.

Most prior studies focused on generic Y-TZP or a single commercial product, neglecting potential variations in stabilizer composition (Y_2_O_3_, CeO_2_), sintering protocols, and grain size among clinically available brands. This study directly compares three widely used Y-TZP systems to identify material-specific aging resistance, offering clinicians evidence-based selection criteria. Many studies employ lab-pressed or machined specimens that poorly replicate CAD/CAM-fabricated prostheses [13,14,15]. In contrast, we use industrially sintered blocks processed via CAD/CAM—identical to chairside workflows—ensuring translational validity.

Therefore, the primary objective of this study was to comprehensively evaluate the low-temperature degradation (LTD) behavior of three widely used commercial Y-TZP dental ceramics—**Cercon^®^, e.max^®^ ZirCAD, and Vita In-ceram^®^ YZ**—under clinically relevant aging conditions. To simulate long-term oral environmental effects, specimens were immersed in 4% acetic acid at 80 °C for 0, 7, 14, and 28 days, providing critical insights into time-dependent phase stability and chemical solubility. Importantly, all samples were prepared using CAD/CAM manufacturing processes to closely replicate real-world dental laboratory workflows, thereby ensuring the direct clinical applicability of the findings. Furthermore, this study investigated the impact of two clinically essential surface treatments—sandblasting and polishing—on LTD resistance, addressing a key controversy in prosthodontic research regarding their long-term effects on zirconia’s structural integrity.

## 2. Materials and Methods

All of the reagents were commercially obtained and used without further purification. Acetic acid (≥99.7%) was purchased from Sigma-Aldrich (Sigma-Aldrich Chemie GmbH, Steinheim, Germany; Merck KGaA, Darmstadt, Germany). Distilled water (H_2_O) was prepared in the laboratory.

**Figure 1:**
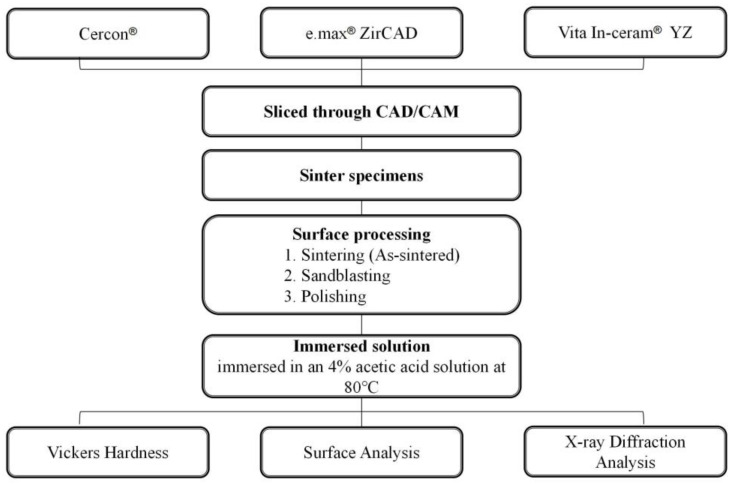
Flowchart of the immersion experiment setup.

The study design is schematically illustrated in Figure 1. The pre-sintered materials of the yttria-stabilized zirconia specimens in this study were purchased from three commercial manufacturers: Cercon^®^: Cercon (DeguDent) (ZrO_2_ > 92%, Y_2_O_3_ 5%, HfO_2_ < 2%, other oxides < 1%); e.max^®^ ZirCAD: IPS e.max ZirCAD for inLab^®^ (Ivoclar) (ZrO_2_ > 92%, Y_2_O_3_ 5%, HfO_2_ < 3%, Al_2_O_3_ + Si_2_O_3_ < 1%); Vita In-ceram^®^ YZ: In-ceram^®^ 2000 YZ CUBES for inLab^®^ (Vita) (ZrO_2_ > 92%, Y_2_O_3_ 4–6%, HfO_2_ < 1–5%, other oxides < 1%).

### 2.1. Specimen Preparation

The commercial zirconia porcelain blocks were sliced into specimens (20 mm × 5 mm × 3 mm, length × width × thickness) utilizing CAD/CAM technology. All specimens were placed in a high-temperature sintering furnace for sintering, according to the manufacturer’s recommendations. The sintering temperature requirements for **Cercon^®^**, **e.max^®^ ZirCAD**, and **Vita In-ceram^®^ YZ** were 1350 °C for 6.5 h, 1500 °C for 7.5 h, and 1530 °C for 7.5 h, respectively. We randomly divided the specimens into six groups, each containing five specimens.

### 2.2. Surface Processing

The specimens were divided into three groups according to different surface treatments, namely, the sintering group, sandblasting group, and polishing group. Specifically, the sintering group consisted of “as-sintered” specimens without additional treatment. In the sandblasting group, the specimen surfaces were sprayed with 120 μm alumina circulating sand under 4 bars of pressure. In the polishing group, the specimens were polished with 600#, 800#, 1200#, 1500#, and 2000# water sandpaper. Subsequently, the samples were polished with 3 μm and 1 μm diamond paste.

### 2.3. Low-Temperature Degradation Tests

Low-temperature degradation was evaluated according to the chemical immersion method used in a previous study. The three groups of 3Y-TZP specimens were immersed in acetic acid solution (4% (*V*/*V*)) at 80 °C. The immersion time was extended from 7 days (168 h) to 14 days (336 h) and 28 days (672 h), according to the reported method [16]. In addition, the mechanical properties of the three dental zirconia ceramics were investigated after LTD.

### 2.4. Vickers Hardness Measurements

The Vickers hardness was determined using the indentation technique (FM-300e type c, FUTURE-TECH CORP, Kawasaki, Japan). An indentation load of 2 kg was applied for 15 s.

### 2.5. Scanning Electron Microscopy Analysis

The microstructures of three different surface treatments after accelerated immersion were analyzed under a vacuum environment using a scanning electron microscope (SEM) (JSM-7401F, JEOL, Tokyo, Japan). Prior to SEM examination, the specimens were coated with gold to prevent charge accumulation.

### 2.6. X-Ray Diffraction Analysis

The progress of *t*→*m* transformation and phase identification were analyzed with X-ray diffraction analysis (XRD, MiniFlex II, Rigaku, Japan) using Cu-Kα radiation (*λ* = 1.5418 Å). XRD analysis was conducted in the 2*θ* range of 25–37° with a step size of 0.02° and a scanning speed of 1 °/min. The XRD accelerating voltage and emission current were 30 kV and 15 mA, respectively. The monoclinic/tetragonal phase ratio, *X_m_*, was determined using the following equation, introduced by Garvie and Nicholson [17]:

(1) $$X_{m}=\frac{I_{m}\left(\bar{1}11\right)+I_{m}\left(111\right)}{I_{m}\left(\bar{1}11\right)+I_{m}\left(111\right)+I_{t}(111)}$$

where $I_{m}\left(111\right)$, $I_{m}\left(\bar{1}11\right)$, and $I_{t}(111)$ denote the relative intensities of the monoclinic (111) (2*θ* = 31.2°), monoclinic $\left(\bar{1}11\right)$ (2*θ* = 28°), and tetragonal (2*θ* = 30°) phases, respectively [18].

### 2.7. Crystalline Grain Size Calculations

The crystalline grain size was calculated according to the intercept method of ASTM E112-2013 (R2021) [19] as follows: crystalline grain size = (total length measured/number of grains passed) × (1/magnification).

### 2.8. Transformed Zone Depth

The total monoclinic phase thickness was obtained using the XRD method originally presented in the reports [20], i.e., the transformed zone depth (TZD), which was calculated as follows:

(2) $$TZD=\frac{sin\;\theta }{2\;\mu }\;ln\frac{1}{1-Xm}$$

where *θ* = 15° is the angle of reflection, the absorption coefficient is *µ* = 0.0642, and *X_m_* denotes the relative monoclinic phase content obtained from XRD analysis.

## 3. Results

### 3.1. XRD Analysis

**Figure 2:**
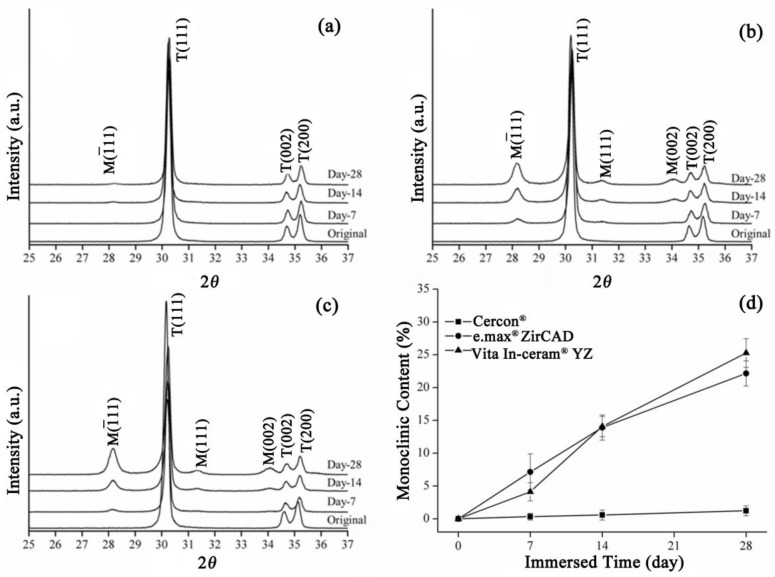
Sintering group: (**a**) XRD results after LTD tests for **Cercon^®^**. (**b**) XRD results after LTD tests for **e.max^®^ ZirCAD**. (**c**) XRD results after LTD tests for **Vita In-ceram^®^ YZ**. (**d**) Monoclinic phase content (%) after LTD tests.

The XRD patterns of the sintering group are shown in Figure 2. The **Cercon^®^** specimens exhibited a minute amount of monoclinic phases after 28 days of immersion (Figure 2a). Figure 2b,c show the XRD patterns of **e.max^®^ ZirCAD** and **Vita In-ceram^®^ YZ**, respectively. After soaking for 28 days, diffraction peaks appeared at about 28° and 31.5°, and with increased soaking time. The diffraction peak intensity also increased, indicating that the monoclinic crystal phase increased with soaking time.

The monoclinic phase content in the sintering group zirconia samples was calculated according to the Garvie and Nicolson formula (Equation (1)). The relative amounts of monoclinic zirconia in the specimens detected using XRD are shown in Figure 2d. The monoclinic phase content of the three commercial zirconia ceramics increased with prolonged immersion time, and the **e.max^®^ ZirCAD** specimens had a monoclinic phase fraction similar to that of **Vita In-ceram^®^ YZ** after 28 days of immersion, with values of 22% and 25%, respectively. However, the phase transformation of **Cercon^®^** was relatively low, at only 2%.

**Figure 3:**
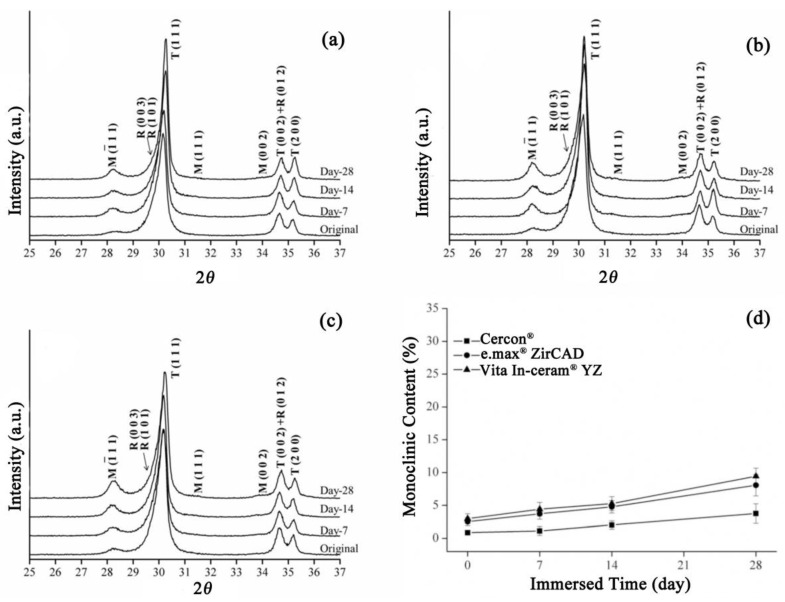
Sandblasting group: (**a**) XRD results after LTD tests for **Cercon^®^**. (**b**) XRD results after LTD tests for **e.max^®^ ZirCAD**. (**c**) XRD results after LTD tests for **Vita In-ceram^®^ YZ**. (**d**) Monoclinic phase content (%) after the LTD tests.

Residual stress is known to be one of the causes of phase transformation of zirconia. Therefore, three types of commercial zirconia were sandblasted to investigate the effect of sandblasting on the degradation of zirconia. Figure 3 shows the XRD patterns of the **Cercon^®^**, **e.max^®^ ZirCAD**, and **Vita In-ceram^®^ YZ** zirconia ceramic blocks after sandblasting. Sandblasting on the surface of porcelain blocks can cause phase change from the tetragonal phase (*t*-ZrO_2_) to the monoclinic phase (*m*-ZrO_2_), as well as the rhombohedral phase (*r*-ZrO_2_) [21]. In general, the rhombohedral phase serves as an intermediate in the *t*→*m* transformation, which can act as a barrier to further initiate the transformation of (*t*-ZrO_2_). After grinding, residual stress remained on the surface of the material, causing *r*→*m* transformation. As shown in Figure 3a–c, the test results had a very obvious monoclinic phase diffraction peak after sandblasting, with an obviously asymmetric diffraction peak at T (111), which mainly included tetragonal phase and rhombohedral crystal phases, indicating that residual stress was generated after sandblasting. Zinelis et al. [22] also determined that sandblasting could produce more monoclinic and rhombohedral phases than grinding and polishing.

As shown in Figure 3a, the T (111) diffraction peak of the **Cercon^®^** sandblasting test specimen gradually shifted from asymmetry to symmetry with soaking time, indicating that the rhombohedral phase gradually disappeared with prolonged soaking time. According to the theory that rhombohedral crystal phases will change to monoclinic crystal phases after stress relief [21], it can be concluded that the rhombohedral crystal phase of the **Cercon^®^** sandblasting test piece would change to the monoclinic crystal phase with soaking time. At the T (111) diffraction peak in the XRD patterns, the strength of the test specimen of **e.max^®^ ZirCAD** (Figure 3b) and **Vita In-ceram^®^ YZ** (Figure 3c) sandblasting group gradually decreased with immersion time. Moreover, the asymmetric diffraction peak shape was still maintained, and the strength of the rhombohedral phase was not reduced, indicating that the rhombohedral phase was still present in the material, which did not gradually transform into a monoclinic crystal phase with immersion but directly transformed from the tetragonal crystal phase.

As shown in Figure 3d, after soaking on the 28th day, the monoclinic crystal contents of **e.max^®^ ZirCAD** and **Vita In-ceram^®^ YZ** were about 8% and 9%, respectively. However, the phase variable of **Cercon^®^** was relatively low, at only 3%. We speculated that the phase variable of the sandblasting group was lower than that of the sintering group, possibly due to the large amount of residual stress on the surface caused by sandblasting, making the tetragonal phase more stable [7].

**Figure 4:**
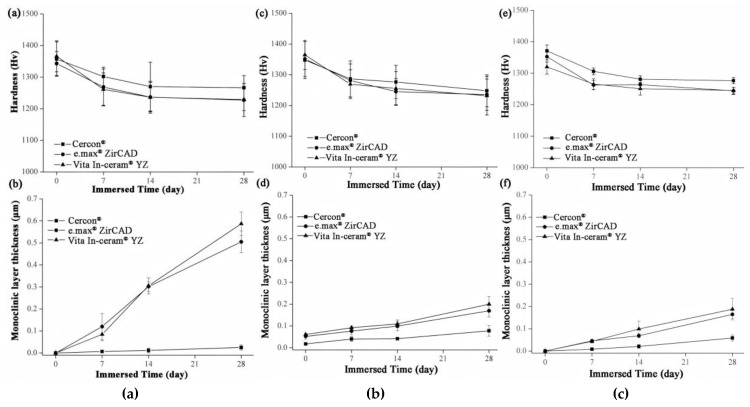
Polishing group: (**a**) XRD results after LTD tests for **Cercon^®^**. (**b**) XRD results after LTD tests for **e.max^®^ ZirCAD**. (**c**) XRD results after LTD tests for **Vita In-ceram^®^ YZ**. (**d**) Monoclinic phase content (%) after LTD tests.

Slight asymmetry was observed in the T(111) diffraction peak of the XRD pattern of the **Cercon^®^** (Figure 4a), **e.max^®^ ZirCAD** (Figure 4b), and **Vita In-ceram^®^ YZ** (Figure 4c) polishing groups, mainly due to the production of the trace rhombohedral phase, which was caused by residual stress as a result of grinding and polishing. As shown in Figure 4d, the monoclinic phases in the **e.max^®^ ZirCAD** and **Vita In-ceram^®^ YZ** polishing groups after soaking for 28 days accounted for about 7% and 8%, respectively. Papanagiotou et al. [23] demonstrated that a layer of stress on the surface could be removed when the surface polishing reached 1 µm, which had the effect of slowing down phase transition.

### 3.2. SEM Investigation

**Figure 5:**
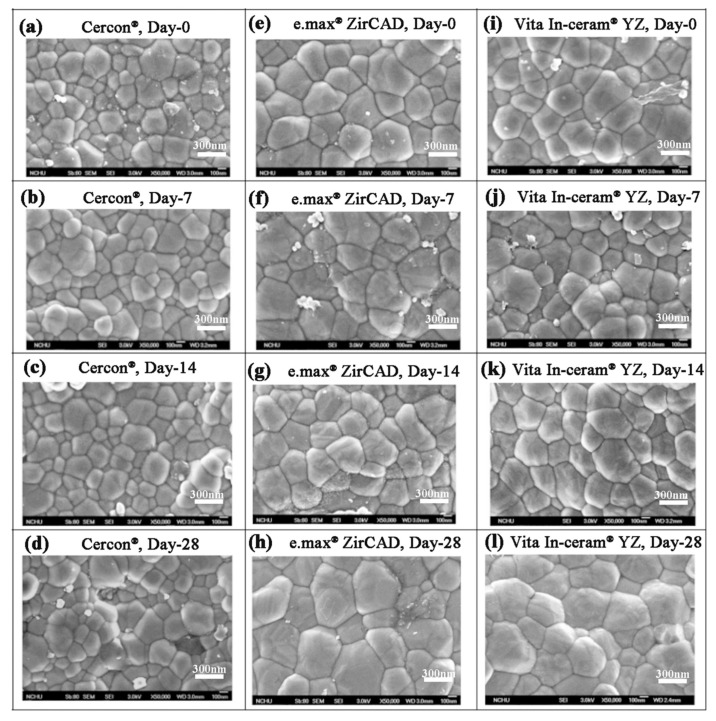
Surface structures of the sintered specimens observed by SEM after the LTD tests: (**a**) **Cercon^®^**, day 0; (**b**) **Cercon^®^**, day 7; (**c**) **Cercon^®^**, day 14; (**d**) **Cercon^®^**, day 28; (**e**) **e.max^®^ ZirCAD**, day 0; (**f**) **e.max^®^ ZirCAD**, day 7; (**g**) **e.max^®^ ZirCAD**, day 14; (**h**) **e.max^®^ ZirCAD**, day 28; (**i**) **Vita In-ceram^®^ YZ**, day 0; (**j**) **Vita In-ceram^®^ YZ**, day 7; (**k**) **Vita In-ceram^®^ YZ**, day 14; (**l**) **Vita In-ceram^®^ YZ**, day 28.

Lughi and Sergo [7] revealed that three main factors influenced the LTD behavior of zirconia ceramics: (i) stabilizer type and content, (ii) stress, and (iii) grain size. In this study, 5% Y_2_O_3_ was used as a stabilizer for the three commercial surface untreated zirconia ceramics. Therefore, the most possible influential factor of phase transformation for these three commercial zirconia ceramic blocks was the difference in grain size. The SEM images of **Cercon^®^**, **e.max^®^ ZirCAD**, and **Vita In-ceram^®^ YZ** shown in Figure 5 indicated that the grain size of the **Cercon^®^** ceramic block was smaller than that of the other two brands. According to the ASTM E112 intercept distance method, the grain sizes of the sintered **Cercon^®^**, **e.max^®^ ZirCAD**, and **Vita In-ceram^®^ YZ** ceramic blocks were 281 ± 9, 461 ± 11, and 495 ± 6 nm, respectively.

**Figure 6:**
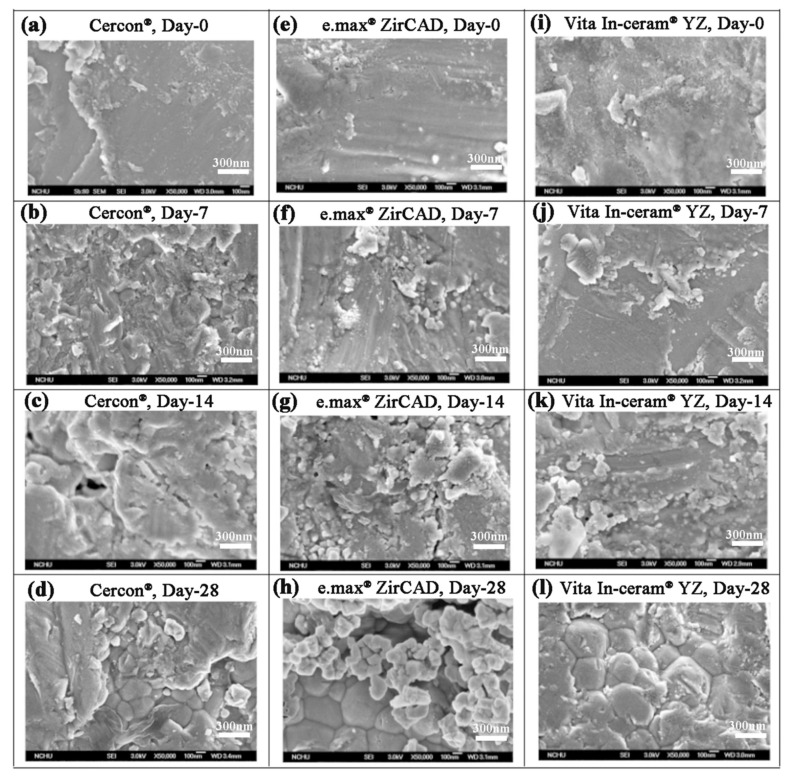
Surface structures of the sandblasting group specimens observed by SEM after LTD testing: (**a**) **Cercon^®^**, day 0; (**b**) **Cercon^®^**, day 7; (**c**) **Cercon^®^**, day 14; (**d**) **Cercon^®^**, day 28; (**e**) **e.max^®^ ZirCAD**, day 0; (**f**) **e.max^®^ ZirCAD**, day 7; (**g**) **e.max^®^ ZirCAD**, day 14; (**h**) **e.max^®^ ZirCAD**, day 28; (**i**) **Vita In-ceram^®^ YZ**, day 0; (**j**) **Vita In-ceram^®^ YZ**, day 7; (**k**) **Vita In-ceram^®^ YZ**, day 14; (**l**) **Vita In-ceram^®^ YZ**, day 28.

Kim et al. [24] showed that sandblasting could make the surface of the material rough and easy to crack. After soaking, phase transition would easily extend from the crack to the inside of the material. When the stress on the surface was relieved and the grain inside started to undergo phase transition, the cracks increased, and the surface of the material started to peel off. As shown in Figure 6, the SEM images of **Cercon^®^** demonstrated that some crack lines were present on the surface of the sandblasted test piece after soaking for 7 days (Figure 6b). When the soaking time was extended to 28 days, some peeling of the material surface was observed (Figure 6d). However, surface cracks, peeling phenomenon, and even the internal grains were observed for **e.max^®^ ZirCAD** (Figure 6g) and **Vita In-ceram^®^ YZ** (Figure 6k) after soaking for 14 days. We inferred that the phase transition rate was fast and that the phase variable increased with stress removal after soaking [25].

**Figure 7:**
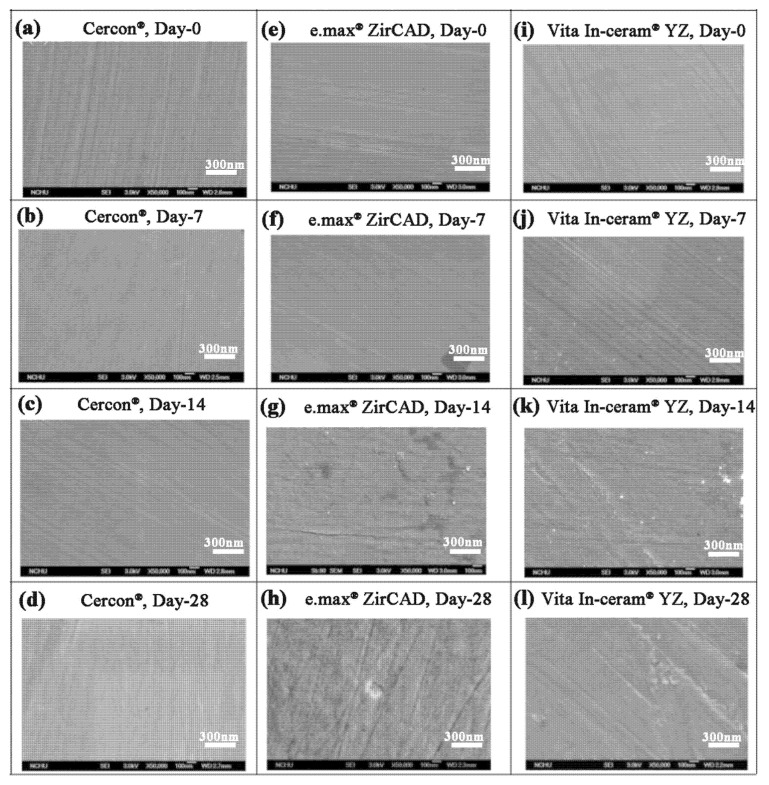
Surface structures of the polishing group specimens observed by SEM after LTD testing: (**a**) **Cercon^®^**, day 0; (**b**) **Cercon^®^**, day 7; (**c**) **Cercon^®^**, day 14; (**d**) **Cercon^®^**, day 28; (**e**) **e.max^®^ ZirCAD**, day 0; (**f**) **e.max^®^ ZirCAD**, day 7; (**g**) **e.max^®^ ZirCAD**, day 14; (**h**) **e.max^®^ ZirCAD**, day 28; (**i**) **Vita In-ceram^®^ YZ**, day 0; (**j**) **Vita In-ceram^®^ YZ**, day 7; (**k**) **Vita In-ceram^®^ YZ**, day 14; (**l**) **Vita In-ceram^®^ YZ**, day 28.

The SEM images of the **Cercon^®^, e.max^®^ ZirCAD,** and **Vita In-ceram^®^ YZ** polishing group samples are shown in Figure 7. The **Cercon^®^** porcelain pieces had a slightly rough surface after soaking. The **Vita In-ceram^®^ YZ** porcelain blocks started to peel from the grinding scratch after soaking for 14 days (Figure 7k), and cracks appeared on the surface until day 28 (Figure 7l) because the scratch was the area where phase transition and stress concentrations were most obvious. However, the **e.max^®^ ZirCAD** porcelain pieces contained grain protrusions on day 28 due to phase changes on the surface of the test sheet (Figure 7h). The main reason was volume expansion resulting from the transformation of the tetragonal crystal phase to the monoclinic crystal phase.

### 3.3. Microhardness Analysis

**Figure 8:**
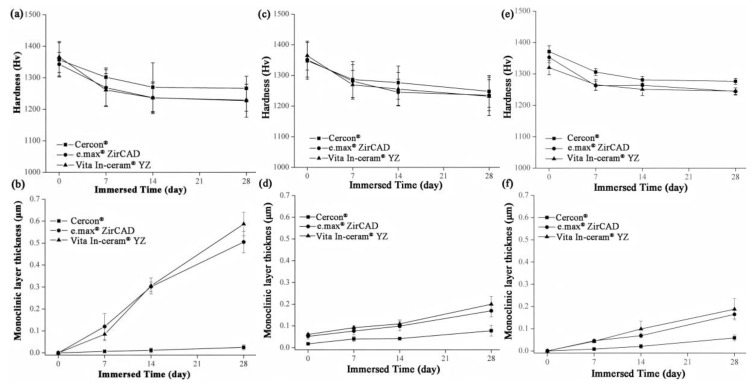
(**a**) Hardness analysis of sintered specimens after LTD tests. (**b**) Phase transformed zone depth of sintered specimens after LTD tests. (**c**) Hardness analysis of sandblasting group specimens after LTD tests. (**d**) phase transformed zone depth of the sandblasting group specimens after LTD testing. (**e**) Hardness analysis of the polishing group specimens after LTD tests. (**f**) Phase transformed zone depth of polishing group specimens after LTD tests.

The microhardness analysis results of the sintered specimens for **Cercon^®^**, **e.max^®^ ZirCAD,** and **Vita In-ceram^®^ YZ** are shown in Figure 8a. Before immersion, the microhardness of the three commercial zirconia ceramics was close to ~1350 Hv. After 28 days of immersion, the microhardness of **Cercon^®^** was slightly higher than that of the other zirconia ceramics, with a value of 1267 ± 39 Hv. The microhardness values of **e.max^®^ ZirCAD** and **Vita In-ceram^®^ YZ** were 1229 ± 52 and 1227 ± 35 Hv, respectively, and the results were all within the margin of error. The results showed that the microhardness of the three commercial zirconia ceramics would decrease after immersion, and the reduction rate was almost the same.

Figure 8c shows the microhardness of the sandblasting test sheet after immersion. After 28 days of immersion, the microhardness of **Cercon^®^** was slightly higher than that of the other two zirconia samples, with a value of 1247 ± 51 Hv. The microhardness values of **e.max^®^ ZirCAD** and **Vita In-ceram^®^ YZ** were 1235 ± 50 and 1231 ± 62 Hv, respectively.

Figure 8e shows the microhardness of the grinding and polishing test samples after soaking. After soaking for 28 days, the microhardness of **Cercon^®^** was slightly higher than that of the other two zirconia samples, with a value of 1276 ± 9 Hv. The microhardness values of **e.max^®^ ZirCAD** and **Vita In-ceram^®^ YZ** were 1245 ± 10 and 1244 ± 11 Hv, respectively.

## 4. Discussion

**Figure 9:**
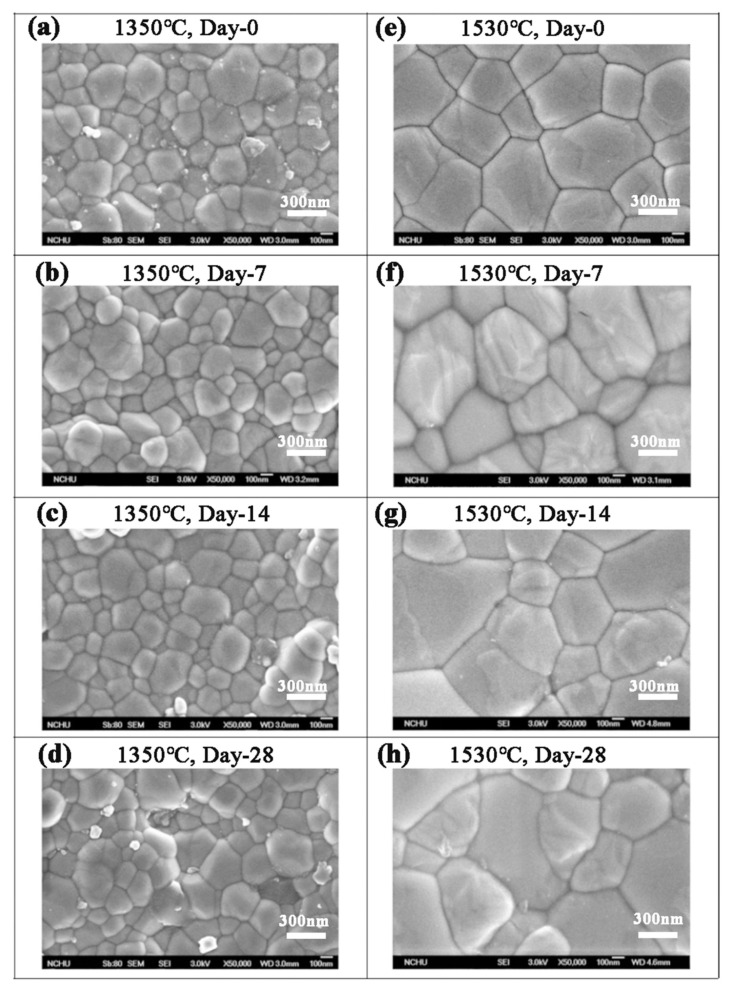
SEM surface observations of the **Cercon^®^** sintered specimens at different temperatures after low-temperature degradation testing: (**a**) 1350 °C sintered specimens, immersed for 0 days; (**b**) 1350 °C sintered specimens, immersed for 7 days; (**c**) 1350 °C sintered specimens, immersed for 14 days; (**d**) 1350 °C sintered specimens, immersed for 28 days; (**e**) 1530 °C sintered specimens, immersed for 0 days; (**f**) 1530 °C sintered specimens, immersed for 7 days; (**g**) 1530 °C sintered specimens, immersed for 14 days; (**h**) 1530 °C sintered specimens, immersed for 28 days.

According to the SEM images in Figure 5, the grain size of **Cercon^®^** was smaller than that of the other two brands. The phase transformation of **Cercon^®^** was relatively low because the **Cercon^®^** porcelain blocks were sintered at a lower temperature. To confirm the experimental results in which the phase variable was related to the grain size, the sintering temperature of **Cercon^®^** increased from the original temperature of 1350 °C to 1530 °C. As shown in Figure 9, after sintering at 1530 °C, the grain size increased from 281 ± 9 to 536 ± 8 nm, according to SEM analysis.

**Figure 10:**
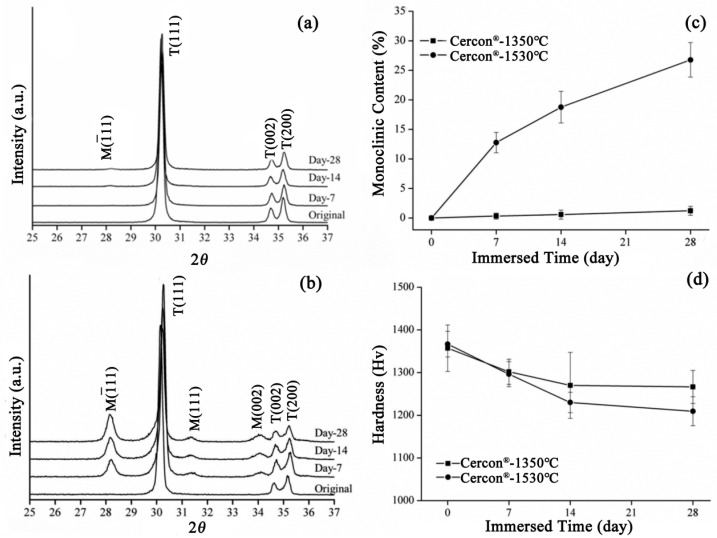
(**a**) XRD results for the **Cercon^®^** samples sintered at 1350 °C after LTD testing. (**b**) XRD results for **Cercon^®^** sintering at 1530 °C after LTD testing. (**c**) Monoclinic phase content (%) after LTD testing of the **Cercon^®^** specimens. (**d**) Microhardness analysis of the **Cercon^®^** sintered specimens after LTD tests.

The XRD patterns of the **Cercon^®^** specimens sintered at 1350 °C and 1530 °C after soaking for 28 days are depicted in Figure 10a and Figure 10b, respectively. The diffraction peaks of the **Cercon^®^** specimens sintered at 1530 °C were obviously generated at about 28° and 31.5°, and the intensity of diffraction peaks also increased with prolonged soaking time, indicating that the monocline phase increased with soaking time. Figure 10c shows the phase transition of the **Cercon^®^** specimens sintered at 1350 °C and 1530 °C after accelerated immersion. We clearly observed that the test specimen sintered at 1530 °C demonstrated a significant increase in phase variable, and the monoclinic phase content was 26% after 28 days of immersion. In terms of microhardness, the hardness value of the **Cercon^®^** specimens sintered at 1530 °C was also slightly lower than that of the original specimen sintered at 1350 °C, at about 1209 ± 33 Hv (Figure 10d).

A literature review by Lughi and Sergo [7] indicated that a grain size < 300 nm was more stable, which could inhibit low-temperature degradation. Schmauder and Schubert [21] proposed that stress caused by the thermal expansion anisotropy of large grains was greater than that of the small grains; thus, the stability of the large grains was poor. According to the literature [26], under the same low-temperature degradation test conditions, the grain size was larger, and the phase transition was faster. Therefore, the differences in the LTD tests of the three commercial zirconia ceramics in this study were mainly due to the differences in grain size caused by different sintering temperatures.

The significantly enhanced resistance to LTD observed in **Cercon^®^** compared to **e.max^®^ ZirCAD** and **Vita In-ceram^®^ YZ** can be primarily attributed to its distinct microstructural characteristics. Our experimental results demonstrate that Cercon^®^ maintains a small grain size (281 ± 9 nm) even after 28 days of immersion, in contrast to the coarser grains observed in **e.max^®^ ZirCAD** (461 ± 11 nm) and **Vita In-ceram^®^ YZ** (495 ± 6 nm). The submicron grain structure of Cercon^®^ creates greater mechanical constraint against the tetragonal-to-monoclinic phase transformation. The increased grain boundary density effectively raises the activation energy required for phase transformation. A smaller grain size reduced the critical transformation zone volume, as predicted by classical phase transformation theory [20,27]. These microstructural advantages collectively contribute to **Cercon^®^**’s superior LTD resistance, as evidenced by its minimal monoclinic phase content (2%) compared to **e.max^®^ ZirCAD** (22%) and **Vita In-ceram^®^ YZ** (25%) after prolonged immersion. The findings highlight the critical importance of grain size control in developing durable dental zirconia ceramics for long-term clinical applications.

According to an investigation by Nono [28], the hardness decreased with the transformation of the tetragonal phase into the monoclinic phase. Furthermore, the hardness was lower with greater monoclinic phase content. Lange et al. [29] believed that the reason for the phase transition of zirconia was that OH^−^ in water occupied the oxygen vacancies and reacted with Y_2_O_3_ to produce Y(OH)_3_ dissolution, which reduced the concentration of the stabilizer. Moreover, Yoshimura et al. [30] believed that OH^−^ in water occupied the oxygen vacancy and reacted with Zr to form Zr-OH, resulting in phase transition. Phase transitions always occur from the surface of the material, gradually extending inward. According to Equation (2) [20], the monoclinic phase transformed the zone depth of the sintering group after 28 days of immersion, as shown in Figure 8b. The TZD values of **Cercon^®^**, **e.max^®^ ZirCAD,** and **Vita In-ceram^®^ YZ** were 0.02 ± 0.01, 0.5 ± 0.04, and 0.58 ± 0.05 µm, respectively. The phase transformation increased with immersion time; however, the thickness of the monoclinic phase layer on the surface was still shallow, which had limited influence relative to the taper needle for the hardness test. In addition to the monoclinic phase pressed to the surface layer, the taper needle for hardness testing also pressed the overall tetragonal phase. The hardness of the specimens with more phase transformation was lower because the taper needle pressed more into the monoclinic phase region of the surface layer; therefore, the amount of phase transformation only slightly affected the hardness value.

The TZD curves of the sandblasting group specimens for **Cercon^®^**, **e.max^®^ ZirCAD,** and **Vita In-ceram^®^ YZ** are shown in Figure 8d. After soaking for 28 days, the TZD values of **Cercon^®^**, **e.max^®^ ZirCAD,** and **Vita In-ceram^®^ YZ** were 0.07 ± 0.02, 0.16 ± 0.02, and 0.20 ± 0.03 µm, respectively. The phase variable of the sandblasting group was less than that of the sintering group, possibly due to the large amount of residual stress caused by sandblasting, which slowed down the conversion rate of the tetragonal phase to the monoclinic phase [31]. Furthermore, as shown in Figure 8f, the TZD values of the polishing group specimens for **Cercon^®^**, **e.max^®^ ZirCAD,** and **Vita In-ceram^®^ YZ** were 0.05 ± 0.01, 0.16 ± 0.01, and 0.18 ± 0.04 µm, respectively. The phase variable of the polishing group was less than that of the sintering and sandblasting groups. Kim, Covel, Guess, Rekow, and Zhang [24] attempted sandblasting and grinding polishing treatment on the surface of Y-TZP after CAD/CAM cutting, which degraded at a low temperature of 122 °C. The research results showed that the phase variable of the grinding and polishing test pieces was lower than that of the sandblasting test piece, which was also consistent with the experimental results. In comparison, the reduction in phase variables could be suppressed by surface treatment.

The findings of this study have significant clinical relevance for restorative dentistry. The marked differences in LTD resistance among commercial Y-TZP brands underscore the importance of material-specific treatment protocols. Clinicians should prioritize **Cercon^®^** for cases requiring long-term stability, particularly in patients with acidic oral environments or parafunctional habits. The superior performance of polished surfaces over sandblasted ones suggests that final polishing should be mandatory in clinical workflows, while sandblasting should involve strict parameter control (≤50 μm Al_2_O_3_ at <2 bar) when unavoidable. These evidence-based recommendations can substantially improve the 10-year survival rates of zirconia prostheses.

However, several limitations highlight critical research needs. While the accelerated aging model is standardized, it cannot fully replicate the complex oral environment where mechanical fatigue, thermal cycling, and biofilm interactions synergistically affect degradation. Future studies should employ multifactorial aging chambers that combine chemical, thermal, and mechanical stressors. Additionally, the rapid evolution of dental zirconia (e.g., 5Y-TZP, multilayer systems) necessitates expanded material evaluations. Most urgently, clinical trials tracking phase transformation in vivo using Raman spectroscopy or synchrotron XRD are needed to validate laboratory predictions. Addressing these gaps will enable development of truly bio-stable zirconia formulations and AI-powered lifetime prediction tools for personalized prosthesis planning. This study establishes a critical foundation for precision prosthodontics, where material selection and handling are tailored to individual patient risk factors. By bridging current knowledge gaps, we can achieve the ultimate goal: lifelong zirconia restorations that can withstand the harsh oral environment without degradation.

## 5. Conclusions

The primary conclusions of this study are as follows:

1. After immersion, the commercial dental zirconia monoclinic phase fractions of **e.max^®^ ZirCAD** and **Vita In-ceram^®^ YZ** were similar, with values of 22% and 25%, respectively. However, the monoclinic phase fraction of **Cercon^®^** was only 2%. The grain size of Cercon^®^ was smaller than that of **e.max^®^ ZirCAD** and **Vita In-ceram^®^ YZ.** It was found that a finer grain structure (<300 nm) inhibited the t→m transformation.
2. The differences in commercial dental zirconia in the LTD test were mainly related to the grain size. The products with low sintering temperatures had smaller grains, which could effectively inhibit the occurrence of phase transformation and reduce the LTD of zirconia.
3. Commercial dental zirconia could inhibit LTD after surface treatment and immersion, and the inhibitory effect of grinding polishing was better than that of sandblasting.
4. In our future work, we will further explore flexural strength, fracture toughness, wear resistance, surface roughness analysis, and material adhesion after modifications. In addition, the biological cell experiments on these materials after processing will be part of the subsequent work to explore their impact in the biological environment.

## Data Availability

The original contributions presented in this study are included in the article. Further inquiries can be directed to the corresponding author.

## Abbreviations

The following abbreviations are used in this manuscript:

LTD	Low-temperature degradation
SEM	Scanning electron microscope
TZD	Transformed zone depth
XRD	X-ray diffraction
Y-TZP	Yttria-stabilized tetragonal zirconia polycrystal
